# Combined repetitive inhalant endotoxin and collagen-induced arthritis drive inflammatory lung disease and arthritis severity in a testosterone-dependent manner

**DOI:** 10.1152/ajplung.00221.2023

**Published:** 2023-12-12

**Authors:** Jill A. Poole, Geoffrey M. Thiele, Elizabeth Ramler, Amy J. Nelson, Michael J. Duryee, Aaron D. Schwab, Angela Gleason, Carlos D. Hunter, Rohit Gaurav, Todd A. Wyatt, Bryant R. England, Ted R. Mikuls

**Affiliations:** ^1^Division of Allergy & Immunology, Department of Internal Medicine, University of Nebraska Medical Center, Omaha, Nebraska, United States; ^2^Veterans Affairs Nebraska-Western Iowa Health Care System, Research Service, Omaha, Nebraska, United States; ^3^Division of Rheumatology & Immunology, Department of Internal Medicine, University of Nebraska Medical Center, Omaha, Nebraska, United States; ^4^Department of Environmental, Agricultural & Occupational Health, College of Public Health, University of Nebraska Medical Center, Omaha, Nebraska, United States; ^5^Division of Pulmonary Critical Care & Sleep, Department of Internal Medicine, University of Nebraska Medical Center, Omaha, Nebraska, United States

**Keywords:** autoimmunity, inflammation, interstitial lung disease, sex differences, testosterone

## Abstract

Respiratory-related diseases are a leading cause of death in rheumatoid arthritis (RA) and are disproportionately higher in men, which may be attributable to environmental risk factors. Animal studies have demonstrated potentiated autoimmunity, arthritis, and profibrotic/inflammatory lung disease with a combination of airborne exposures and collagen-induced arthritis (CIA). This study aimed to determine whether hormone-dependent differences explained these observations. Arthritis-prone male intact and castrated DBA/1J mice received intranasal inhalation of lipopolysaccharide (LPS) daily for 5 wk and CIA induction. Arthritis scores and serum pentraxin-2 levels were increased in castrated versus intact mice. In contrast, airway cell influx, lung tissue infiltrates, and lung levels of proinflammatory and profibrotic markers (C5a, IL-33, and matrix metalloproteinases) were reduced in castrated versus intact mice. CIA + LPS-induced lung histopathology changes and the expression of lung autoantigens including malondialdehyde acetaldehyde (MAA)- and citrulline (CIT)-modified proteins and vimentin were reduced in castrated animals. There were no differences in serum anti-MAA or anti-CIT protein antibody (ACPA) levels or serum pentraxin levels between groups. Testosterone replacement led to a reversal of several lung inflammatory/profibrotic endpoints noted earlier in castrated male CIA + LPS-treated mice with testosterone supplementation promoting neutrophil influx, MAA expression, and TNF-α, IL-6, and MMP-9. These findings imply that testosterone contributes to lung and arthritis inflammatory responses following CIA + LPS coexposure, but not to systemic autoantibody responses. The CIA + LPS model provides a paradigm for investigations focused on the mechanistic underpinnings for epidemiologic and phenotypic sex differences in RA-related lung disease.

**NEW & NOTEWORTHY** Our study shows that testosterone acts as a key immunomodulatory hormone contributing to critical features of rheumatoid arthritis (RA)-associated lung disease in the setting of airborne endotoxin (lipopolysaccharide; LPS) exposures and concomitant arthritis induction in mice. The exaggerated airway inflammation observed following combined exposures in male mice was accompanied by increases in profibrotic mediators, netosis, and increased expression of lung autoantigens, all relevant to the pathogenesis of lung disease in arthritis.

## Graphical abstract

**Figure:**
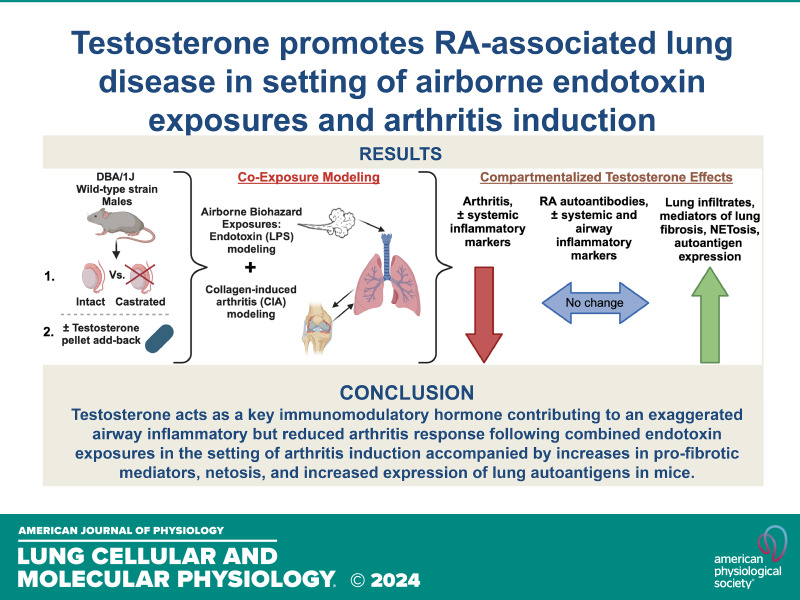

## INTRODUCTION

Rheumatoid arthritis (RA) is a systemic autoimmune disease predominately characterized by inflammatory arthritis that is often associated with a number of extra-articular manifestations including interstitial lung disease (ILD), chronic bronchitis, chronic obstructive pulmonary disease (1), pulmonary nodules, and pleural diseases (2). Respiratory-related conditions are among the leading causes of death in men and women with RA (3, 4) with ILD clinically impacting up to 10% of patients with RA and subclinically affecting up to 40% (5, 6). Importantly, ILD is substantially more common among men with RA despite RA being two to three times more common in women. Several airborne exposures have been implicated as risk factors in RA and RA-associated lung disease with differences in the frequency (or intensity) of these exposures speculated to explain sex-based differences in disease expression (7). Environmental factors implicated in disease development and progression include the well-established risk factor of cigarette smoking as well as high concentration ambient air pollution, military-related exposures (burn pits, organic dusts, and waste disposal), and various workplace environments (agriculture, textile, silica, construction, and mining) (7–11). Among women, the link between occupational and other environmental exposures and RA risk has been less clear than in men (7). Whether differences in the risk of RA and RA-related lung disease are due to variability in environmental exposures or sex-related biological effects has not been elucidated.

It is recognized that male mice have heightened susceptibility to acute lipopolysaccharide (LPS)-induced lung inflammation (12) and that inhalant LPS-induced bone loss was attenuated with estradiol supplementation, both in male mice as well as ovariectomized female mice (13). In contrast, it has been demonstrated that ovariectomy did not alter airway inflammatory response to LPS in female mice, but castration prevented exaggerated LPS-induced airway inflammatory response in male mice (14). More recent modeling strategies combining repetitive airborne exposures including LPS (15) or agriculture organic dust extracts with arthritis (collagen-induced arthritis, CIA) demonstrated the potentiation of inflammatory joint disease, increased airway inflammatory cell influx, and increased deposition of lung extracellular matrix (ECM) (16, 17). Combined modeling also resulted in increased expression of autoantigens in lung tissues including both citrullinated (CIT)- and malondialdehyde acetaldehyde (MAA)-modified lung proteins, with associated increases in circulating anti-MAA autoantibodies and anti-CIT protein antibody (ACPA) (16–18). ACPA responses are highly specific in RA whereas anti-MAA autoantibodies have been identified as novel biomarkers of RA-associated lung disease (19). Moreover, the animal modeling strategy combining inhaled LPS or organic dust extracts with CIA demonstrated that male mice were profoundly more susceptible to most effects of coexposure as compared with female mice, particularly notable for increased arthritis severity and lung inflammatory/profibrotic responses (17), suggesting that this modeling approach could provide important insight into sex-based differences seen in RA and RA-ILD.

The objective of this study was to quantify the effects of gonadectomy on lung and arthritis outcomes following exposure to the combination of repetitive inhalant LPS and CIA in arthritis-susceptible male mice (i.e., DBA/1J strain). Moreover, we sought to examine whether differences observed in arthritis, autoantibody production, and lung inflammatory/profibrotic consequences would be attenuated with specific hormone add-back administration. The overarching goal of this study was to understand the potential mechanistic role of sex hormones in the dynamic interplay of lung inflammation with arthritis and systemic autoimmunity that characterize RA.

## MATERIALS AND METHODS

### Animal Modeling

We used an established 5-wk exposure model (16) whereby mice were randomized to 1 of 2 treatment groups including Sham (saline injection/saline inhalation) or CIA plus repetitive inhalant LPS (CIA + LPS, CIA injection/LPS inhalation). Arthritis-prone, wild-type DBA/1J intact and castrated male mice, and intact and oophorectomized female mice between 6 and 8 wk of age were purchased from The Jackson Laboratory (Bar Harbor, ME) and allowed to acclimate for 1 wk before initiation of experiments. DBA/1J mice are not otherwise genetically modified, are commonly utilized in experimental RA modeling studies, and have been utilized by our research group in prior coexposure modeling studies of inhalant environmental exposures with systemic arthritis induction (16, 17). Note that testicular castration and oophorectomy were performed at The Jackson Laboratory at age 3–4 wk. CIA was induced as per the Chondrex protocol (Chondrex, Inc., Redmond, WA) (17). Airway inflammation was induced using repetitive intranasal inhalation of LPS (16) whereby mice were lightly sedated under isoflurane and received treatment with either 50 µL of sterile saline or LPS (100 ng in 50 µL sterile saline) from *Escherichia coli* (O55:B5; Sigma, St. Louis, MO) daily for 5 wk (weekends excluded). Weights were recorded throughout the treatment period. Animals were euthanized 5 h following the final exposure.

For testosterone add-back studies, slow-release, 60-day testosterone [5α-dihydrotestosterone, 15 mg, concentration based upon work by others (20)] and placebo pellets (Innovative Research of America, Sarasota, FL) were implanted subcutaneously in the lateral neck between the ear and shoulder by trocar following administration of anesthesia with isoflurane inhalation.

All animal studies are detailed in accordance with the Animal Research Reporting In Vivo Experiments (ARRIVE) guidelines (https://arriveguidelines.org/). Animal procedures were approved by the Institutional Animal Care and Use Committee and were in accordance with the NIH guidelines for the use of rodents.

### Arthritis Evaluation

Mice were assessed weekly for the development of arthritis using the semiquantitative, mouse arthritis scoring system provided by Chondrex (www.chondrex.com). This protocol is based on foot examination with a range of 0 (no inflammation) to 4 (erythema and severe swelling encompassing ankle, foot, and digits).

### Serum Markers

Serum was derived from whole blood collected at the time of euthanasia from the axillary artery. Circulating testosterone and the murine acute phase reactant protein pentraxin-2 were quantified by Quantikine ELISA according to manufacturer’s instructions [minimal detection dose (MDD) of 0.066 ng/mL, BioVendor Laboratory Medicine, RRID:SCR_005143, Asheville, NC and 0.368 ng/mL, R&D Systems, Minneapolis, MN, respectively]. ACPA and anti-MAA (IgG) antibodies were quantified using ELISA with data presented as relative units (RUs) of the specific isotype detected (18, 21, 22). Briefly, human serum albumin, type II collagen, and vimentin were used as substrate antigens for anti-MAA antibody measurement whereas human serum albumin was used as substrate antigen for ACPA quantification.

### Lavage Fluid Cells and Inflammatory Markers

Bronchoalveolar lavage fluid (BALF) was collected using 3 × 1 mL of phosphate-buffered saline (PBS). Total cell numbers from the combined recovered lavage were enumerated using a BioRad TC 20 cell counter and differential cell counts were determined from cytospin-prepared slides (Cytopro cytocentrifuge, ELITech Group, Logan, UT) stained with DiffQuick (Siemens, Newark, DE). From cell-free supernatant of the first lavage fraction, tumor necrosis factor-alpha (TNF-α), interleukin (IL)-6, and murine neutrophil chemokines (CXCL1 and CXCL2) were quantitated by ELISA (R&D Systems, MDD of 7.2, 1.8, 2.0, and 1.5 pg/mL, respectively.) These analytes have been implicated in airborne exposure-induced lung inflammation and RA-associated lung disease (16). Specific calcium-binding S100 protein S100A8, which is associated with impaired matrix remodeling in RA (23) and formation of neutrophil extracellular traps (NETs) (24), was quantitated by ELISA (Abcam, Waltham, MA; MDD of 50 pg/mL).

### Lung Cell Staining and Flow Cytometry

Following vascular perfusion and removal of BALF, harvested lungs (1/2 of the right lung) were subjected to an automated dissociation procedure using a gentleMACS Dissociator instrument (Miltenyi Biotech, Auburn, CA) (16). The viability of total lung cells was assessed by trypan blue exclusion and a LIVE/DEAD Fixable Blue Dead Cell Stain Kit (Invitrogen, Carlsbad, CA). Less than one percent of gated cells were not viable, with no differences by treatment group (data not shown). Lung cells from each animal were incubated with CD16/32 (Fc Block, BD Biosciences, San Jose, CA) to minimize nonspecific antibody staining, and then stained with monoclonal antibodies (mAb) directed against rat anti-mouse CD45 (clone: 30-F11; eBiosciences), CD11b (clone: M1/70; Biolegend), Ly6G (clone 1A8; Biolegend), CD11c (clone: N418; Invitrogen), CD4 fluorescein-5-isothiocyanate (FITC) (clone RM4-5, BD Biosciences), CD8a PE (clone 53-6.7, BD Biosciences), CD19 allophycocyanin (APC) eFluor 780 (clone 1D3, Invitrogen), and hamster anti-mouse CD3e PE-Cy7 (clone 145-2C11, BD Biosciences). Cells were acquired on a BD LSRII Yellow/Green cytometer configured with 355-nm, 405-nm, 488-nm, 561-nm, and 633-nm lasers. Postacquisition, data were exported and stored using the flow cytometry standard (FCS) 3.1 format and analyzed using FlowJo software version 10.8 (FlowJo, RRID:SCR_008520, Ashland, OR). The gating strategy for Ly6G^+^ neutrophils, CD11c^+^CD11b^lo^ alveolar macrophages, CD11c^+^CD11b^+^ activated macrophages, CD11c^int^CD11b^+^ transitioning/recruited monocytes-macrophages, CD11c^−^CD11b^+^ monocytes, CD3^+^CD4^+^ T cells, CD3^+^CD8^+^ T cells, CD19^+^ B cells was a previously published (16, 18, 25) and depicted in Supplemental Fig. S1. The percentage of all respective cell populations was determined from live CD45^+^ lung leukocytes after excluding debris and doublets. This percentage was multiplied by the respective total lung cell numbers to determine specific cell population numbers for each animal.

### Analytes from Lung Homogenates

Lung tissue homogenates were prepared by homogenizing lung samples (one-half of the right lung) in 500 μL of sterile PBS. Matrix metalloproteinases (MMPs) and metallopeptidase inhibitor (TIMP-1) levels were determined by Magnetic Luminex Assay (R&D), with MDDs of 1276.5 pg/mL for MMP-8, 33.9 pg/mL for MMP-9, and 24.7 pg/mL for TIMP-1. Levels of the alarmin IL-33 (R&D, MDD of 14.3 pg/mL) and complement component/anaphylatoxin C5a (R&D, MDD of 15.6 pg/mL) were determined by individual ELISAs. Levels of lung double-stranded (ds) DNA, which are associated with NET production (26), were quantitated by NanoDrop One spectrophotometer (Thermo Scientific).

### Lung Histopathology and Autoantigens

Following lavage and vascular perfusion, left lungs were excised and inflated to 15 cmH_2_O pressure with 10% formalin (Fisher Scientific, Fair Lawn, NJ) for 24 h to preserve pulmonary architecture (16). The fixed-left lung lobes were then placed into cassettes ventral side down, embedded in paraffin, cut (4–5 μm) at midpoint sections to include regions of both large and small airways as well as blood vessels, and stained with hematoxylin and eosin (H&E) (or preserved for subsequent immunohistochemistry) by the Tissue Science Core Facility at the Department of Pathology and Microbiology at the University of Nebraska Medical Center. H&E-stained slides of the entire lung section from each animal were reviewed and semiquantitatively scored by an expert pathologist (27) blinded to treatment conditions (16, 27). This scoring system (scored 0 to 4) evaluates the spectrum of inflammatory changes for alveolar and bronchiolar compartments with higher scores indicating greater inflammation. The number of ectopic cellular aggregates was also assessed.

Lung sections were also stained with anti-myeloperoxidase (MPO, 1:100, Cat. No. ab9535, Lot No. GR331736-4, Abcam), anti-neutrophil elastase (ELA2, 1:100, Cat. No. NBP2-66972, Lot No. H01122, Novus, Centennial, CO), and anti-histone H2B (1:100, Cat. No. ab52484, Lot No. GR298234-1, Abcam). Cross absorbed (H + L) donkey anti-rabbit Alexa Fluor (AF) 555 (Cat. No. A31572, Lot No. 1806147), goat anti-rabbit AF 555 (Thermo Fisher Scientific Cat. No. A32732, RRID:AB_2633281, Lot No. RJ243416), and goat-anti-mouse AF 488 (Cat. No. A232723, Lot No. RJ243426) (Invitrogen, Rockford, IL) were used at 1:100 dilution as secondary antibodies. Slides were mounted with VECTASHIELD Antifade Mounting Medium with DAPI (4′,6-diamidino-2-phenylindole; to identify nuclei) (Cat. No. H-1200, Lot No. ZJ0808, Newark, CA) and visualized under a Zeiss fluorescent microscope. MPO^+^ neutrophils, neutrophil elastase, and histone H2B staining were quantified by Image J FIJI plugin (16, 18) (NIH, Bethesda, MD).

Lung autoantigens (MAA and CIT) and vimentin were stained (18). Vimentin is an ECM protein that is increased in inflammatory lung diseases and targeted by post-translational modifications as previously demonstrated by colocalization with MAA and CIT in RA-ILD (19). Lung sections were stained with Cy5 rabbit anti-vimentin (Bioss, Woburn, MA, 1:100), Zenon AF 594 label (Invitrogen, Carlsbad, CA), rabbit polyclonal IgG antibody to MAA, or a mouse monoclonal anti-peptidyl-citrulline antibody (clone F95 IgMκ, Millipore Sigma, Burlington, MA). Detection of the F95 antibody was done using an AF 488 AffiniPure donkey anti-mouse IgM, µ chain-specific antibody (Jackson Immunoresearch, West Grove, PA). DAPI (4′,6-diamidino-2-phenylindole; to identify nuclei) was added and samples were sealed with Fluormount-G (Southern Biotech, Birmingham, AL). Fluorochrome detections were done using a Revolve fluorescent microscope (ECHO, San Diego, CA). Images were quantified using ImageJ and colocalization was performed using the Image J (RRID:SCR_003070) FIJI plugin Coloc 2 as previously described (16, 18).

### Statistical Analysis

Power and sample-size calculations were extrapolated from a previous study (16), whereby we calculated a sample size of *n* = 7 in each group, to achieve 80% power at the 0.05 level of significance to detect a 40% difference in total cellular influx assuming a mean (SD) of 2.02 × 10^6^ (0.52) of the CIA + LPS male mice. Data are presented as the mean with standard deviation (SD). The nonparametric Kruskal–Wallis test was applied, and if *P* value was less than 0.05, the Benjamini and Hochberg (BH) test was applied for multiple comparisons to control the false discovery rate to determine differences between groups (28). A two-way ANOVA for two variables (i.e., treatment groups vs. time point) was applied to determine differences in arthritis severity score. In confocal studies, the FIJI plugin, Coloc 2 in ImageJ was used to quantify the magnitude of colocalization of vimentin, MAA, or CIT using five regions of interest from each image. Pearson’s correlation coefficients were calculated based on the overlap of two colors to generate an *R*^2^ value. *R*^2^ values from the five regions of interest were averaged for each mouse. All statistical analyses were performed using GraphPad Prism (RRID:SCR_002798, v. 9.0.0) software and statistical significance was accepted at a two-sided *P* < 0.05.

## RESULTS

### Combined Modeling of CIA + LPS with Arthritis Outcomes, Weight Changes, and Levels of Serum Testosterone, Pentraxin-2, and Autoantibodies in Intact versus Castrated Male Mice

**Figure 1.**
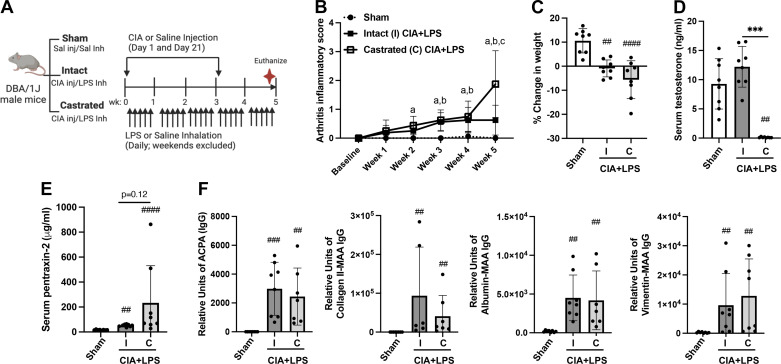
Combined modeling of repetitive lipopolysaccharide (LPS)-induced airway inflammation with collagen-induced arthritis (CIA) with arthritis, weight changes, and serum testosterone, pentraxin-2 and autoantibodies in intact vs. castrated male mice. *A*: schematic of experimental design. Intact (I) and castrated (C) male mice vs. Sham mice received CIA or saline (sal) injection and daily LPS or saline intranasal inhalations (inh) for 5 wk. *B*: line graph depicts means with SD bars of arthritis inflammatory score over time with *P* < 0.05 denoted as sham vs. castrated (“a”) or intact (“b”) CIA + LPS and intact vs. castrated CIA + LPS (“c”) as determined by two-way ANOVA with Benjamini and Hochberg (BH) test for multiple comparisons to control the false discovery rate. Scatterplots depict means with SE bars of the percent change in weight (*C*) and serum levels of testosterone (*D*) and the acute phase reactant protein pentraxin-2 (*E*). *F*: serum levels of IgG anti-citrullinated protein antibody (ACPA) to citrullinated peptides, IgG antibody to malondialdehyde-acetaldehyde (MAA)-modified collagen type II, human serum albumin, and vimentin proteins across treatment groups. Statistical analyses were performed with Kruskal–Wallis and BH test for multiple comparisons test (##*P* < 0.01, ###*P* < 0.001, ####*P* < 0.0001) vs. sham and (***P < 0.001) between groups; *n* = 8 mice/group (and time point). [Image *A* created with a licensed version of BioRender.com.]

In the first set of experiments, wild-type intact versus castrated male mice underwent arthritis induction (CIA) with 5 wk of daily LPS inhalation (CIA + LPS) and were compared with wild-type Sham-treated mice (saline injection/saline inhalation) (schematic, Fig. 1*A*). Throughout the 5-wk experiment, arthritis scores increased for intact and castrated CIA + LPS versus Sham, starting at 2 wk (castrated) and 3 wk (intact) (Fig. 1*B*). At 5 wk, the highest arthritis score was for castrated CIA + LPS versus both intact CIA + LPS (*P* < 0.05) and Sham (*P* < 0.05). Compared with Sham, CIA + LPS treatment resulted in decreased weight over time without significant differences between intact and castrated mice (Fig. 1*C*). Serum testosterone levels were significantly reduced in castrated versus intact CIA + LPS mice (*P* < 0.0001) without significant differences between Sham and intact CIA + LPS mice (Fig. 1*D*). Serum levels of the acute phase reactant pentraxin-2 were increased with CIA + LPS coexposure for both intact and castrated versus Sham. Although quantitatively higher in the castrated versus intact male CIA + LPS mice, this difference in serum pentraxin-2 levels did not achieve statistical significance (*P* = 0.12) (Fig. 1*E*). Whereas serum autoantibodies including ACPA and anti-collagen II-MAA, albumin-MAA, and vimentin-MAA antibodies were increased with CIA + LPS in both intact and castrated male mice versus Sham, there were no differences in these serum autoantibodies between intact versus castrated mice (Fig. 1*F*).

### CIA + LPS Coexposure-Induced Lung Cell Influx and Proinflammatory/Profibrotic Marker Release Were Reduced in Castrated (vs. Intact) Male Mice

**Figure 2.**
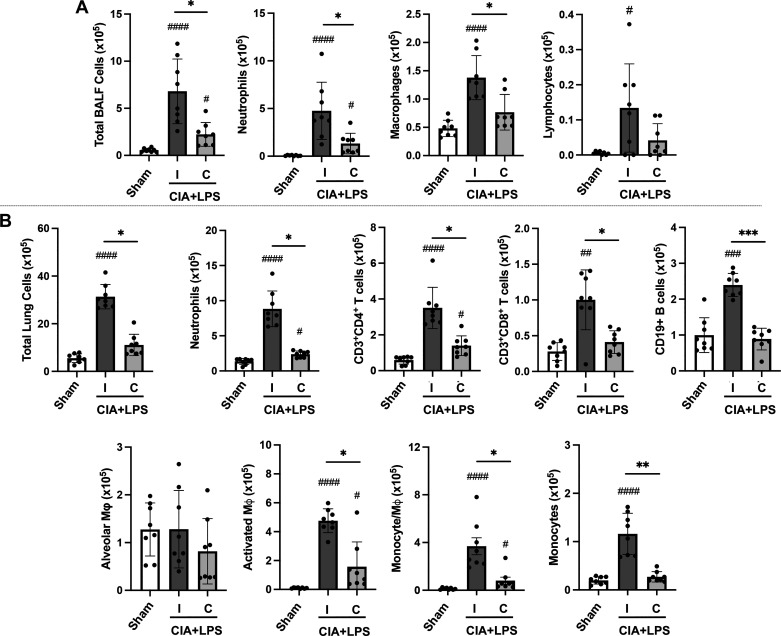
Collagen-induced arthritis (CIA) + lipopolysaccharide (LPS) coexposure-induced lung cell influx is reduced in castrated male mice. Scatterplots with bars depict means with SD. *A*: total cells, neutrophils, macrophages, and lymphocytes enumerated from bronchoalveolar lavage fluid (BALF). *B*: lung cell infiltrates determined by flow cytometry on live CD45^+^ cells after exclusion of debris and doublets with lung cell % populations multiplied by total lug cells enumerated from lung sample. Cells defined as CD11c^−^Ly6G^+^ neutrophils, CD3^+^CD4^+^ T cells, CD3^+^CD8^+^ T cells, CD19^+^ B cells, CD11c^+^CD11b^lo^ alveolar macrophages (Mɸ), CD11c^+^CD11b^+^ activated Mɸ, CD11c^int^CD11b^hi^ transitioning monocyte-Mɸ, and CD11c^−^CD11b^hi^ monocytes. Gating strategy is depicted in Supplemental Fig. S1. Statistical analyses were performed with Kruskal–Wallis test with Benjamini and Hochberg (BH) test for multiple comparisons to control the false discovery rate (#*P* < 0.05, ##*P* < 0.01, ###*P* < 0.001, ####*P* < 0.0001) vs. Sham and (*P < 0.05, **P < 0.01, ***P < 0.001) between groups; *n* = 8 mice/group.

In contrast to increased arthritis in castrated versus intact mice coexposed to CIA + LPS, CIA + LPS-induced influx of BALF total cells, neutrophils, and macrophages were reduced following castration with a similar trend observed for lymphocytes (Fig. 2*A*). Correspondingly, total cell infiltrates in lung tissues were increased with CIA + LPS coexposure in both intact and castrated mice versus Sham, but this difference was again significantly attenuated following castration (*P* < 0.0001 vs. intact mice) (Fig. 2*B*). Similar patterns were observed for specific cell subpopulations including Ly6G^+^ neutrophils, CD3^+^CD4^+^ T cells, CD3^+^CD8^+^ T cells, CD19^+^ B cells, activated CD11c^+^CD11b^+^ macrophages, transitioning/recruited CD11c^int^CD11b^+^ monocyte-macrophages, and CD11c^−^CD11b^+^ monocytes (Fig. 2*B*). In contrast to total cells and most cell subpopulations, there were no differences in the number of resting alveolar macrophages (CD11c^+^CD11b^lo^) across groups (Sham, CIA + LPS intact or castrated).

**Figure 3.**
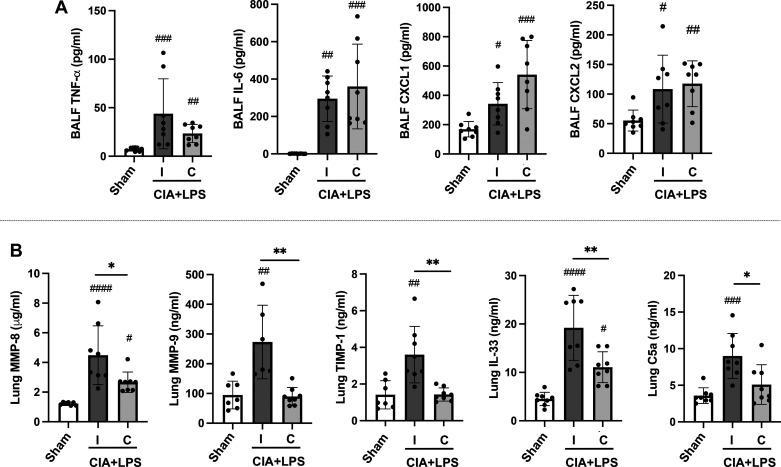
Combined collagen-induced arthritis (CIA) + lipopolysaccharide (LPS) exposure-induced airway inflammatory mediator release and levels of lung tissue extracellular matrix proteins, alarmin IL-33, and complement C5a are reduced in castrated male mice. Scatter plots with bars depict means with SD. *A*: bronchoalveolar lavage fluid (BALF) levels of proinflammatory cytokines [tumor necrosis factor-alpha (TNF-α), interleukin (IL-6)] and neutrophil chemoattractants (CXCL1, CXCL2). *B*: lung tissue levels of matrix metalloproteinases (MMPs), metallopeptidase inhibitor (TIMP-1), IL-33, and complement component C5a. Statistical analyses were performed with Kruskal–Wallis and Benjamini and Hochberg (BH) test for multiple comparisons test (#*P* < 0.05, ##*P* < 0.01, ###*P* < 0.001) vs. sham; and (*P < 0.05, **P < 0.01) between groups; *n* = 8 mice/group.

CIA + LPS coexposure also resulted in increased BALF levels of TNF-α, IL-6, CXCL1, and CXCL2 in both intact and castrated mice versus Sham in the absence of significant differences between the intact and castrated mice (Fig. 3*A*). In contrast, differences in mediators involved in wound repair/fibrosis between intact and castrated CIA + LPS treated mice were demonstrated in lung tissue homogenates. Namely, in intact mice, CIA + LPS exposure increased the levels of ECM proteins (MMP-8, MMP-9, TIMP-1), alarmin IL-33, and complement component C5a versus both Sham and castrated mice (Fig. 3*B*). Compared with intact mice, tissue concentrations for all of these proteins were significantly attenuated following castration with only MMP-8 and IL-33 levels higher in castrated versus Sham mice.

### Combined CIA + LPS Exposure-Induced Lung Histopathology, Neutrophil Infiltrates, Netosis Markers, and Lung Autoantigens Were Reduced in Castrated (vs. Intact) Male Mice

**Figure 4.**
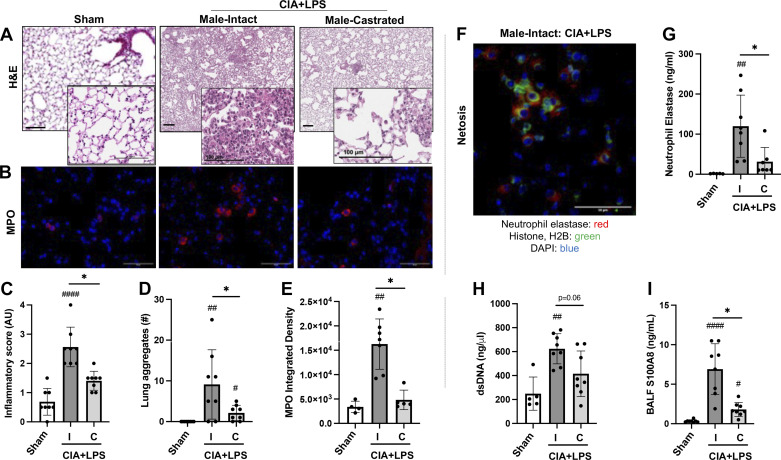
Collagen-induced arthritis (CIA) + lipopolysaccharide (LPS) coexposure-induced lung histopathology, neutrophil infiltrates, and netosis markers are reduced in castrated male mice. Representative hematoxylin and eosin (H&E, *A*) and confocal microscopy image of myeloperoxidase (*B*; MPO, red and nuclei; DAPI, blue)-stained lung section images from each treatment group. Line bar is 100 μm. Scatterplots depict means with SD of semiquantitative lung inflammatory score (*C*) and number of lung aggregates enumerated (*D*) from H&E-stained sections and integrated density of MPO staining (*E*). *F*: representative image of “netosis” evident by neutrophil elastase (red), histone/H2B (green), nuclei (DAPI, blue) with integrated density of neutrophil elastase staining (*G*). Quantification of lung tissue dsDNA (*H*) and bronchoalveolar lavage fluid (BALF) levels of S100A8 (*I*), all markers of netosis. Statistical analyses were performed with Kruskal–Wallis test with Benjamini and Hochberg (BH) test for multiple comparisons to control the false discovery rate (#*P* < 0.05, ##*P* < 0.01, ###*P* < 0.001) vs. sham; and (*P < 0.05) between groups. *n* = 4–8 mice/group. Note that the H&E Sham staining was conducted separately from the CIA + LPS-treated sections but analyzed collectively. All other images were collected at the same time under the same conditions.

Lung sections of Sham, CIA + LPS intact, and CIA + LPS castrated mice were next evaluated for histopathological changes by H&E (Fig. 4*A*) and for MPO^+^ neutrophils (Fig. 4*B*). CIA + LPS coexposure induced increases in semiquantitative inflammatory scores (Fig. 4*C*) and the number of ectopic cellular aggregates versus Sham, both of which were reduced in castrated versus intact mice (Fig. 4*D*). Lung MPO^+^ neutrophils were increased in CIA + LPS intact mice versus Sham (Fig. 4*E*), a finding that was again significantly attenuated with castration. Correspondingly, markers of NETosis, a program for the formation of neutrophil extracellular traps (NETs) consisting of chromatin and neutrophil granules (29), were prominent in intact mice following CIA + LPS treatment (Fig. 4, *F–I*). There were small foci of NETs marked by colocalization of neutrophil elastase with histone H2B staining in the intact CIA + LPS male mice (Fig. 4*F*). To quantify NETosis, neutrophil elastase (Fig. 4*G*) and dsDNA (Fig. 4*H*) lung tissue levels were measured and found increased in the CIA + LPS intact mice versus both Sham, differences that were attenuated in castrated mice. BALF levels of S100A8 were increased with CIA + LPS in both intact and castrated mice versus Sham, but this response was significantly reduced (*P* < 0.05) in castrated versus intact animals (Fig. 4*I*).

**Figure 5.**
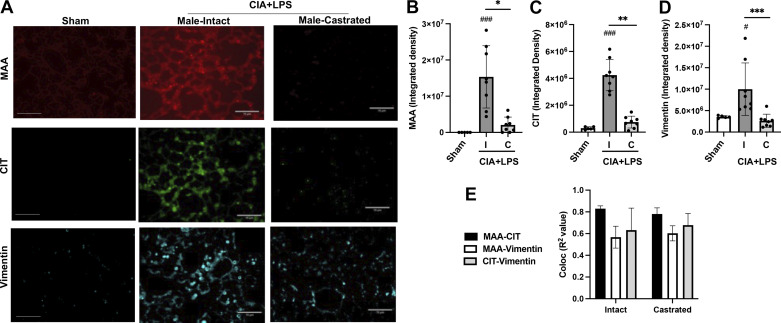
Combined collagen-induced arthritis (CIA) + lipopolysaccharide (LPS) exposure-induced lung citrulline (CIT) and malondialdehyde acetaldehyde (MAA) autoantigens and vimentin expression are reduced in castrated male mice. *A*: representative confocal microscopy images of lung tissue from treatment groups stained for malondialdehyde-acetaldehyde (MAA, red) and citrulline (CIT, green) modified proteins and vimentin (teal). Line scale denotes 100 μm. All images were collected at the same time under the same conditions. Scatterplots depict means with SD of the integrated density of MAA- (*B*) and CIT-modified (*C*) proteins and (*D*) vimentin quantified per each mouse. *E*: *R*^2^ values (correlation coefficient) demonstrating colocalization of MAA-CIT-vimentin from lung tissues. Statistical analyses were performed with Kruskal–Wallis test with Benjamini and Hochberg (BH) test for multiple comparisons to control the false discovery rate (#*P* < 0.05, ###*P* < 0.001) vs. sham and (*P < 0.05, **P < 0.01, ***P < 0.001) between groups; *n* = 8 mice/group.

Lung tissues were also stained for CIT- and MAA-modified antigens as well as vimentin. CIT- and MAA-modified proteins and vimentin were significantly increased in CIA + LPS intact versus Sham mice (Fig. 5, *A–D*). These differences were again significantly attenuated in castrated mice with only MAA values being higher than Sham following castration. Colocalization of autoantigens and vimentin was calculated with Pearson correlation coefficients (*R*^2^ values). CIT and MAA modifications strongly and similarly colocalized in lung tissues of CIA + LPS intact (*R*^2^ value of 0.83) and castrated (*R*^2^ value of 0.78) mice (Fig. 5*E*). Likewise, there were similar degrees of colocalization of MAA-vimentin and CIT-vimentin with CIA + LPS intact (*R*^2^ values of 0.57 and 0.63, respectively) and castrated (*R*^2^ values of 0.52 and 0.65, respectively) mice.

### Effects of Testosterone Add-Back in Intact and Castrated Male Mice in the Setting of CIA + LPS Coexposure Modeling

**Figure 6.**
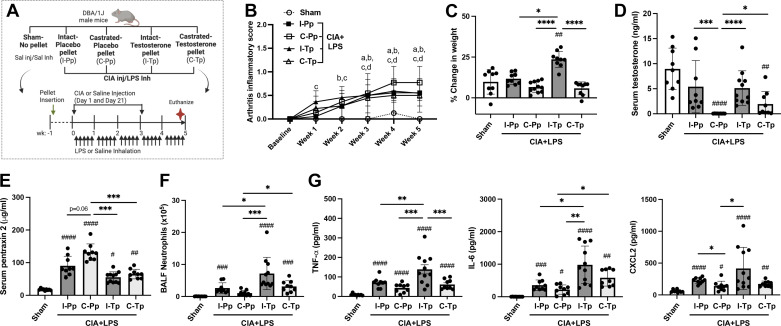
Arthritis, weight changes, serum testosterone, and pentraxin-2 levels, and airway neutrophil influx and inflammatory mediator release in testosterone add-back studies of intact and castrated male mice in the setting of collagen-induced arthritis (CIA) + lipopolysaccharide (LPS) coexposure modeling. *A*: schematic of experimental design. Intact (I) and castrated (C) male mice received either placebo (P) or testosterone (T) pellet (p) followed by combined CIA + LPS exposures. *B*: arthritis inflammatory score over time with *P* < 0.05 denoted as “a”(I-Pp), “b”(C-Pp), “c” (I-Tp), “d” (C-Tp) vs. Sham. The percent change in weight (*C*), serum testosterone (*D*) and pentraxin-2 (*E*) levels, bronchoalveolar lavage fluid (BALF) neutrophils (*F*), and BALF inflammatory mediator levels (*G*) across treatment groups are depicted by scatter dot plots with means ± SD. Statistical analyses were performed with Kruskal–Wallis test with Benjamini and Hochberg (BH) test for multiple comparisons to control the false discovery rate (#*P* < 0.05, ##*P* < 0.01, ###*P* < 0.001, ####*P* < 0.0001) vs. sham and (*P < 0.05, **P < 0.01, ***P < 0.001, ****P < 0.0001) between groups; *n* = 9 (Sham, I-Pp, C-Pp, C-Tp) and *n* = 11 (I-Tp) mice/group. [Image *A* created with a licensed version of BioRender.com.]

Intact versus castrated male mice received either the slow-release placebo-pellet (Pp) or testosterone pellet (Tp) 1 wk before CIA + LPS coexposures with comparisons to Sham mice (no pellet, saline injection/saline inhalation) (schematic, Fig. 6*A*). Throughout the 5-wk experiment, arthritis scores increased for all treatment groups versus Sham, starting at 1 wk (intact-Tp), 2 wk (castrated-Pp), and 3 wk (intact-Pp and castrated-Tp) (Fig. 6*B*). At 5 wk, the highest arthritis score was for castrated-Pp CIA + LPS mice but there were no significant differences across the treatment groups (*P* > 0.05). There was more weight gain in CIA + LPS intact-Tp mice compared with all other groups (*P* < 0.001) (Fig. 6*C*). Serum testosterone levels were reduced in castrated-Pp versus all other groups (*P* < 0.001). Testosterone levels were increased with hormone add-back (castrated-Tp) versus castrated-Pp (*P* < 0.01), demonstrating the effectiveness of the pellet delivery, noting that testosterone levels were lower in castrated-Tp versus Sham mice (Fig. 6*D*).

Serum levels of pentraxin-2 were increased in all CIA + LPS coexposed mice versus Sham, and this response was increased in castrated-Pp mice versus all other CIA + LPS-pelleted animal groups (*P* = 0.06 vs. intact-Pp and *P* < 0.05 vs. intact-Tp and castrated-Tp) (Fig. 6*E*), consistent with the nonpelleted, CIA + LPS castrated versus intact studies shown in Fig. 1*E*. Moreover, testosterone add-back in the castrated mice reversed these findings with castrated-Tp mice demonstrating significantly lower pentraxin-2 levels than castrated-Pp mice (*P* < 0.001). Similar to findings in the nonpelleted intact/castrated murine studies (Fig. 1*F*), there were also no significant differences in serum autoantibodies among the placebo and testosterone pelleted intact and castrated mice treated with CIA + LPS (Supplemental Table S2).

Consistent with the nonpelleted, intact/castrated studies with CIA + LPS coexposure, total leukocytes and neutrophil counts in BALF were reduced in castrated-Pp mice. Notably, these cell counts were increased in intact-Tp versus intact-Pp, suggesting a potentiated response with additional testosterone delivery (Fig. 6*F* and Supplemental Table S2). CIA + LPS-induced airway macrophage influx was increased in castrated-Pp, intact-Tp, and castrated-Tp versus Sham, and there were no significant differences with lymphocyte influx (Supplemental Table S2). Correspondingly, the highest BALF levels of CIA + LPS-induced pro inflammatory TNF-α, IL-6, and CXCL2 were observed for the intact-Tp treatment group, and this met significance (*P* < 0.05) for TNF-α [vs. castrated-Pp and trended (*P* = 0.06) vs. castrated-Tp], IL-6 (vs. Sham, intact-Pp, castrated-Pp), and CXCL2 (vs. Sham, castrated-Pp) (Fig. 6*G*). Levels of IL-6 but not TNF-α and CXCL2 were increased with testosterone add-back in the castrated mice (castrated-Tp) versus castrated-Pp, consistent with at least a partial reversal of phenotype.

**Figure 7.**
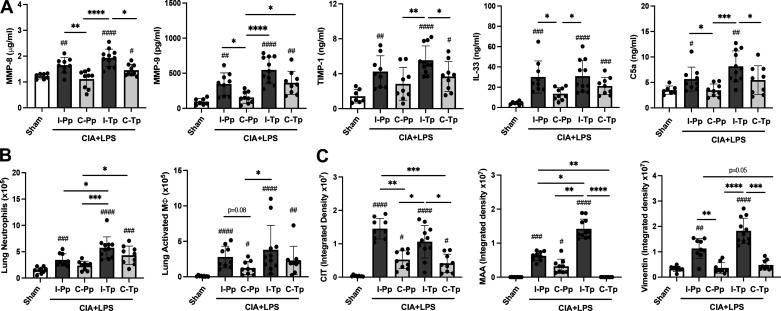
Levels of lung tissue inflammatory/profibrotic mediators, lung cell infiltrates, and expression of lung autoantigens and vimentin following testosterone add-back studies of intact and castrated male mice in the setting of collagen-induced arthritis (CIA) + lipopolysaccharide (LPS) coexposure. Lung levels of matrix metalloproteinase (MMP)-8, MMP-9, metallopeptidase inhibitor (TIMP)-1, interleukin (IL)-33, and C5a (*A*), numbers of lung Ly6G^+^ neutrophils and CD11c^+^CD11b^+^ activated macrophages (*B*), and the integrated density of lung citrulline (CIT)- and malondialdehyde-acetaldehyde (MAA)-modified proteins and vimentin expression (*C*) across treatment groups are depicted by scatter dot plots with means ± SD. Statistical analyses were performed with Kruskal–Wallis test with Benjamini and Hochberg (BH) test for multiple comparisons to control the false discovery rate (#*P* < 0.05, ##*P* < 0.01, ###*P* < 0.001, ####*P* < 0.0001) vs. sham and (*P < 0.05, **P < 0.01, ***P < 0.001, ****P < 0.0001) between groups; *n* = 9 (Sham, I-Pp, C-Pp, C-Tp) and *n* = 11 (I-Tp) mice/group.

Lung tissue levels of ECM proteins, alarmin IL-33, and complement component C5a were also modulated (Fig. 7*A*). Combined CIA + LPS exposure induced increased levels of MMP-8, MMP-9, and TIMP-1 in intact-Pp, intact-Tp, and castrated-Tp but not castrated-Pp versus Sham mice. Testosterone add-back significantly reversed the reduced response to CIA + LPS in castrated mice for MMP-9 but not MMP-8 or TIMP-1. Following observations with the influx of total cells and neutrophils, the highest levels of these ECMs were observed in the intact-Tp mice meeting significance (*P* < 0.05) for MMP-8 (vs. castrated-Pp and castrated-Tp), MMP-9 (vs. castrated-Pp, castrated-Tp), and TIMP-1 (vs. castrated-Pp and castrated-Tp). Similar to observations with ECM proteins aforementioned, CIA + LPS-induced IL-33 levels were increased in intact-Pp, intact-Tp, castrated-Tp mice but not castrated-Pp. Correspondingly, C5a levels were increased only in intact-Pp and intact-Tp but not in intact-Pp or castrated-Pp.

Lung neutrophil counts with CIA + LPS coexposure were increased in intact-Tp versus all other groups except for castrated-Tp mice (Fig. 7*B*). Testosterone add-back reversed the reduced response to CIA + LPS-induced neutrophil infiltrates observed in castrated mice (castrated-Tp > castrated-Pp). CIA + LPS-induced lung-activated macrophages (CD11c^+^CD11b^+^) were increased in the intact-Tp versus castrated-Pp (*P* < 0.05) and trended (*P* = 0.08) for intact-Pp versus castrated-Pp. Although the number of activated macrophages was quantitatively higher with testosterone add-back, the difference with castrated-Pp mice did not reach statistical significance. The remainder of the CIA + LPS-induced lung cell infiltrates as determined by flow cytometry are summarized in Supplemental Table S2.

CIA + LPS exposure-induced lung inflammation (inflammatory score) was increased in all treatment groups versus Sham with reduced scores demonstrated for castrated-Pp versus intact-Pp and intact-Tp (Supplemental Table S2). Next, lung expression of CIT- and MAA-modified proteins and vimentin were increased with combined CIA + LPS exposure for CIT (all treatment groups), MAA (all treatment groups except castrated-Tp), and vimentin (intact-Pp and intact-Tp) versus Sham (Fig. 7*C*). There was potentiation of MAA but not CIT or vimentin expression between intact-Pp versus intact-Tp, and there were no differences between these proteins between castrated-Pp versus castrated-Tp, suggesting that testosterone add-back had no meaningful effect on the lung expression of CIT and vimentin but did have effect on MAA expression.

### CIA + LPS Coexposure Modeling-Induced Arthritis, Serum Autoantibody, and Lung Responses in Ovariectomized versus Intact Female Mice Was Unaltered

Responses to CIA + LPS coexposure between wild-type ovariectomized and intact female mice were also investigated. Whereby there were increases in nearly all endpoints examined with CIA + LPS coexposure versus Sham, there were no differences for these endpoints between ovariectomized and intact female mice (Supplemental Table S1).

## DISCUSSION

Sex differences are well recognized to influence the development of RA, with the disease more frequently affecting women (30) but the extra-articular manifestation of RA-associated lung disease is disproportionately higher in men than women (7). Environmental and occupational exposures implicated in disease development and severity, particularly for RA-lung disease, are important among men but less clear among women (7–11). Recapitulating these human epidemiology observations, male versus female sex differences in RA-lung disease in experimental animal modeling of combining airborne hazard (agriculture organic dust) exposures with arthritis induction have been established (17). Here, we demonstrate that testosterone acts as a key immunomodulatory hormone contributing to critical features of RA-lung disease in the setting of adverse airborne endotoxin (LPS) exposures in CIA mice. Paradoxically, castrated male mice coexposed to CIA + LPS demonstrated increases in arthritis severity accompanied by simultaneous reductions in lung inflammatory cell influx, profibrotic mediator release, lung pathology, NETosis, and lung autoantigen (CIT and MAA) expression. These changes, along with those from hormone add-back studies, suggest that testosterone may yield compartmental effects in men with RA, attenuating arthritis severity while simultaneously promoting proinflammatory cellular and profibrotic changes that characterize RA-ILD.

Although there a several studies evaluating female and male differences in the incidence and prevalence of RA, the explanations for sex differences in RA joint disease development and arthritis severity have been elusive (30) and are unknown in RA-lung disease. In general, estrogens demonstrate dichotomous roles in the immune system by downregulating inflammatory pathways and upregulating immunoglobulin production (31). Estrogen also affects monocyte/macrophage-induced proinflammatory cytokine production, but its inhibitory or stimulatory effect appears to be related to estrogen concentrations (32). We did not identify significant differences in arthritis, serum autoantibody levels, and lung inflammatory indices between oophorectomized and intact female mice coexposed to CIA + LPS.

Instead, profound differences were demonstrated between intact versus castrated male mice treated with combined CIA + LPS exposure. Castrated male mice demonstrated an increase in arthritis severity but strikingly reduced inflammatory lung disease. In contrast to the previously reported systemic “anti-inflammatory” effects of testosterone (33), our observed increase in CIA + LPS induced airway inflammatory responses in intact male mice (vs. castrated mice) is consistent with a previous report of decreased endotoxin (34)-induced airway inflammation in castrated male mice (14). However, reductions observed in airway inflammatory indices following castration in CIA + LPS mice were not universal as there were no differences in the levels of classic proinflammatory cytokines TNF-α and IL-6 or neutrophil chemokines, although testosterone add-back resulted in increased CIA + LPS-induced airway TNF-α and IL-6 release. Importantly, our study shows for the first time that the exaggerated airway inflammation observed in CIA + LPS mice is accompanied by increases in profibrotic mediators (i.e., MMP-8, MMP-9, TIMP-1, IL-33, and C5a), activated CD11c^+^CD11b^+^ macrophages, recruited/transitioning CD11c^int^CD11b^+^ monocytes-macrophages, neutrophils, NETosis, and increased expression of MAA- and CIT-modified proteins, lung autoantigens that have been implicated in the pathogenesis of RA and RA-ILD (19).

Neutrophil extracellular traps (“NETosis”) refer to extracellular DNA extruded from cells, creating webs that release proteases and enzymes promoting bactericidal capacity but that can also injure epithelial and endothelial cells (35). These extracellular traps are also now recognized to release autoantigens associated with several pathologic diseases including RA (35). Although our murine studies suggest a potential role of extracellular traps in the airborne exposure-RA-lung disease axis, the role of extracellular traps in RA-lung disease including RA-ILD has not been well described and future studies are warranted. Whereas resulting lung expression of MAA and CIT occurring in close proximity and colocalized with vimentin were increased with CIA + LPS in intact male mice as compared with castrated male mice, there were no differences in serum autoantibody levels (nor in the testosterone add-back studies). Although the reasons for this discrepancy are unknown, one possibility is that an anticipated increase in autoantibody formation related to increased lung autoantigen expression may be offset by systemic and articular anti-inflammatory effects of testosterone suggested by our findings. Perhaps reflecting the summation of increased arthritis and decreased lung inflammatory/profibrotic processes, serum pentraxin-2 levels (increased in male CIA + LPS mice) were not meaningfully impacted by castration. However, testosterone add-back resulted in reduced pentraxin levels in castrated male CIA + LPS mice, consistent with the “anti-inflammatory” systemic effects of testosterone (33).

Testosterone add-back studies in castrated mice demonstrated reversal of CIA +LPS-induced effects with BALF and lung neutrophil influx, airway release of IL-6, and lung MMP-9 and IL-33, suggesting that these changes were due at least in part to changes in testosterone. However, there were several indices that were not significantly reversed with testosterone add-back in castrated male mice. Changes occurring following castration that were independent of testosterone included BALF levels of TNF-α and CXCL2, lung levels of MMP-8, TIMP-1, and C5a, and lung autoantigen expression as well as lung cellular infiltrates and arthritis severity. There are potential explanations for this lack of reversal with the testosterone add-back studies likely related to dosing, timing, and potentially application route. Moreover, reasons underpinning the finding of lower arthritis severity in the pellet add-back studies and lack of arthritis differences between intact and castrated mice remain unclear but may be related to extra handling of mice accompanying the pellet procedure.

In humans, the so-called minipuberty stage (1–6 mo postnatal) has been suggested as a critical window of programming and imprinting with high testosterone levels directly impacting sex organ development, body mass, and cognitive function (36, 37). Thus, it is possible that the impact of castration resulted in a fixed effect that testosterone cannot reverse. The administration of testosterone in pellet form may also not be reflective of natural testosterone release. In addition, systemic inflammation induced by endotoxin exposure has been shown to reduce testosterone levels in men, albeit the exact mechanisms for this response remain unknown (38).

Testosterone supplementation in the intact mice resulted in potentiation of BALF and lung neutrophil influx, lung recruited/transitioning monocytes-macrophages, lung expression of MAA, and release of TNF-α, IL-6, and MMP-9, despite the lack of significant difference in circulating testosterone concentrations. Testosterone is converted to dihydrotestosterone in most tissues sensitive to androgens including the testes, prostate gland, and muscles. We suspect that muscle mass increased in the testosterone-treated intact males as there was significant weight gain in these male mice versus all other groups. Our observations of enhanced lung consequences following inhalant exposures in the setting of arthritis induction may be an important factor to consider in the management of men with RA on testosterone supplementation. This subgroup of men, particularly those with relevant occupational and other environmental exposures, may be more susceptible to RA-associated lung disease requiring close healthcare surveillance.

Collectively, the current study findings support a striking compartmentalized effect that is dependent on testosterone status in that testosterone skews RA disease phenotype by promoting lung inflammation but appears to be simultaneously protective against more severe joint disease in the setting of airborne exposures and arthritis induction. These compartmentalized effects are highlighted in the lung with testosterone-promoting neutrophil influx, netosis, lung pathology, NETosis, lung pathology, the expression of profibrotic mediators (i.e., MMP-8 and MMP-9) and autoantigen generation (i.e., CIT and MAA), all critical features associated with RA-lung disease that is more common in males versus females. As currently there are no guidelines or algorithms based on sex and/or gender differences (30), further investigation will be essential to better understand the role of sex hormones in disease pathogenesis and to inform the management of RA and RA-related lung disease.

## DATA AVAILABILITY

Data will be made available upon reasonable request.

## SUPPLEMENTAL DATA

10.5281/zenodo.8143607 Supplemental Fig. S1 and Supplemental Tables S1 and S2: https://doi.org/10.5281/zenodo.8143607.

## GRANTS

This work was supported by US Department of Defense Grant PR200793 (to J.A.P. and T.R.M.); National Institute for Occupational Safety and Health (NIOSH) Grant R01OH012045 (to J.A.P.); Department of Veterans Affairs (VA) Grants CSR&D, IK2 CX002203 (to B.R.E.); BLR&D Merit I01 BX004660 (to T.R.M.); BLR&D Merit I01 BX005886 (to T.A.W.); Research Career Scientist Award IK6 BX005962 (to T.A.W.); Rheumatology Research Foundation (to B.R.E.); National Institutes of Health Grants 2U54GM115458 (to T.R.M.), P50 AA030407 (to T.A.W.); Central States Center of Agricultural Safety and Health (CS-CASH)-NIOSH Grant U54 OH010162 (to J.A.P., A.D.S., R.G., and T.A.W.); the University of Nebraska Medical Center (UNMC) Flow Cytometry Research Facility is administrated through the Office of the Vice Chancellor for Research and supported by state funds from the Nebraska Research Initiative (NRI) and The Fred and Pamela Buffett Cancer Center’s National Cancer Institute Cancer Support Grant. Major instrumentation has been provided by the Office of the Vice Chancellor for Research, The University of Nebraska Foundation, the Nebraska Banker’s Fund, and the NIH-National Center for Research Resources (NCRR) Shared Instrument Program.

## DISCLOSURES

J.A.P. has been site investigator for Paraxel, AstraZeneca, GSK with research reagent (anti-IL-33/ST2) for murine research studies from AstraZeneca. B.R.E. has consulted and received research support from Boehringer- Ingelheim. T.R.M. received research support from Horizon Therapeutics and has been a consultant for Horizon, Pfizer, UCB, and Sanofi. None of the other authors has any conflicts of interest, financial or otherwise, to disclose.

## AUTHOR CONTRIBUTIONS

J.A.P., G.M.T., and T.R.M., and conceived and designed research; J.A.P., G.M.T., E.R., A.J.N., M.J.D., A.D.S., A.G., C.D.H., R.G., T.A.W., B.R.E., and T.R.M. performed experiments; J.A.P., G.M.T., E.R., A.J.N., M.J.D., A.D.S., A.G., C.D.H., R.G., T.A.W., B.R.E., and T.R.M. analyzed data; J.A.P., G.M.T., E.R., A.J.N., M.J.D., A.D.S., A.G., C.D.H., R.G., T.A.W., B.R.E., and T.R.M. interpreted results of experiments; J.A.P., G.M.T., E.R., A.J.N., M.J.D., and T.R.M. prepared figures; J.A.P. and T.R.M. drafted manuscript; J.A.P., G.M.T., E.R., A.J.N., M.J.D., A.D.S., A.G., C.D.H., R.G., T.A.W., B.R.E., and T.R.M. edited and revised manuscript; J.A.P., G.M.T., E.R., A.J.N., M.J.D., A.D.S., A.G., C.D.H., R.G., T.A.W., B.R.E., and T.R.M. approved final version of manuscript.
